# Revolutionizing Implantation Studies: Uterine-Specific Models and Advanced Technologies

**DOI:** 10.3390/biom15030450

**Published:** 2025-03-20

**Authors:** Shu-Yun Li, Francesco John DeMayo

**Affiliations:** Reproductive and Developmental Biology Laboratory, National Institute of Environmental Health Sciences, National Institutes of Health, Research Triangle Park, Durham, NC 27709, USA; shuyun.li@nih.gov

**Keywords:** implantation, genetic manipulation, Cre/loxP system, endometrial organoids, uterine receptivity, decidualization, extracellular vesicles, omics technologies

## Abstract

Implantation is a complex and tightly regulated process essential for the establishment of pregnancy. It involves dynamic interactions between a receptive uterus and a competent embryo, orchestrated by ovarian hormones such as estrogen and progesterone. These hormones regulate proliferation, differentiation, and gene expression within the three primary uterine tissue types: myometrium, stroma, and epithelium. Advances in genetic manipulation, particularly the Cre/loxP system, have enabled the in vivo investigation of the role of genes in a uterine compartmental and cell type-specific manner, providing valuable insights into uterine biology during pregnancy and disease. The development of endometrial organoids has further revolutionized implantation research. They mimic the native endometrial structure and function, offering a powerful platform for studying hormonal responses, implantation, and maternal-fetal interactions. Combined with omics technologies, these models have uncovered the molecular mechanisms and signaling pathways that regulate implantation. This review provides a comprehensive overview of uterine-specific genetic tools, endometrial organoids, and omics. We explore how these advancements enhance our understanding of implantation biology, uterine receptivity, and decidualization in reproductive research.

## 1. Introduction

Implantation is a key process for establishing a successful pregnancy and occurs in three distinct stages: apposition, adhesion, and invasion, which establish a maternal-fetal interface that supports the development of the embryo throughout pregnancy [1,2]. Successful implantation requires the synchronized interplay of a receptive uterus and a competent embryo, a process precisely regulated by ovarian hormones, particularly estrogen (essentially 17b-estradiol) and progesterone [3]. During the preovulatory phase of the menstrual cycle, estrogen promotes the proliferation of the endometrial lining [2]. While estrogen is essential for preparing the endometrium, excessive estrogen activity can negatively affect endometrial receptivity [4,5]. Estrogen receptor alpha (ESR1/ERα) plays a significant role during this phase, and its subsequent downregulation by progesterone during the secretory phase is critical for successful implantation [6]. Following ovulation, progesterone drives significant cellular changes within the endometrium, creating a receptive environment for the embryo, known as the “window of implantation (WOI)” [2]. It prepares the uterine epithelium for embryo implantation by suppressing epithelial proliferation before attachment. It also regulates communication between the epithelium and stroma, which is essential for successful embryo attachment and development [7,8]. One of the most critical processes initiated by progesterone is decidualization, which transforms endometrial stromal cells into epithelial-like decidual cells during the mid-secretory phase. This transformation creates a microenvironment suitable for embryo implantation and subsequent placental development in humans [9,10]. Unlike in mice, where decidualization occurs post-implantation, human decidualization is spontaneous and embryo-independent, occurring naturally [11]. This progesterone-regulated process is crucial for pregnancy success, as its disruption can lead to infertility, miscarriage, or uteroplacental disorders [12].

Over the past decade, advances in molecular approaches, omics technologies, and in vitro models, such as endometrial organoids, have enhanced our understanding of uterine biology [13,14,15]. These models, coupled with the development of cell type-specific genetic manipulation tools, such as Cre/loxP mouse models, have provided unprecedented insights into the molecular pathways underlying uterine function during pregnancy [16]. In this review, we aim to provide a comprehensive overview of the tools and technologies that have advanced the study of uterine biology, particularly implantation. We introduce the utility of genetic manipulation in mice, focusing on uterine cell type-specific Cre/loxP models, and explore the application of endometrial organoids in studying implantation, hormonal responses, and maternal-fetal interactions. Additionally, we discuss the contributions of omics technologies in unraveling the complex signaling networks and epigenetic modifications that govern implantation. These emerging tools and approaches continue to reshape our understanding of reproductive biology, offering new opportunities to address fertility challenges and improve maternal health outcomes.

## 2. Genetically Engineered Mice

The use of systemic genetic manipulation has significantly advanced our understanding of the molecular and cellular processes underlying pregnancy, including implantation, decidualization, and placental development [17]. Homeobox A10 (*Hoxa10*) and *Hoxa11* genes are highly expressed in uterine stromal cells and play critical roles in uterine receptivity and stromal remodeling. Deletion of these genes leads to severe reproductive impairments. Specifically, *Hoxa10*-deficient mice exhibit infertility due to implantation failure and early embryo resorption. Morphological analyses reveal that the proximal 25% of the mutant uterus undergoes a homeotic transformation into an oviduct-like structure. Transferring wild-type blastocysts into the mutant uterus does not rescue the implantation defect [18]. *Hoxa11*-deficient females are sterile, but they can produce normal eggs that develop properly post-fertilization when transferred to wild-type surrogate mothers, indicating that uterine receptivity is impaired [19]. Similarly, *Leukemia inhibitory factor* (*Lif*) is a cytokine expressed in the endometrial glands during the receptive phase in mice. *Lif*-deficient females are infertile due to implantation failure, despite producing viable blastocysts capable of implanting and developing when transferred to wild-type pseudopregnant recipients. This indicates that *Lif* is required for successful implantation [20]. In addition to *Lif*, Interleukin 11 (Il-11) signaling mediated by IL-11Rα is crucial for decidualization. Females with hypomorphic IL-11Rα alleles exhibit reduced decidual cell proliferation, resulting in small and degenerating decidua that ultimately fail to support placentation. In IL-11Rα null mice, defective decidualization leads to infertility. IL-11 and IL-11Rα expression peaks during decidualization [21,22].

Steroid hormones are critical regulators of uterine function through receptors such as ESR1 and progesterone receptor (PGR). *Esr1* knockout females are infertile, exhibiting hypoplastic uteri and hyperemic ovaries devoid of corpus luteum, suggesting disrupted ovarian estrogen signaling and uterine responsiveness [23]. *Pgr* knockout mice exhibit an anovulatory phenotype in which mature oocytes become trapped within follicles despite normal follicular growth and oocyte maturation [24]. These phenotypes indicate that systemic knockouts prevent pregnancy establishment and affect multiple tissues, making it unsuitable for studying the specific role of *Esr1* and *Pgr* during pregnancy, as both receptors are expressed across various reproductive tissues with overlapping roles in coordinating systemic and local hormonal responses. Furthermore, systemic knockout models often result in early embryonic lethality, which prevents the study of gene function at later developmental stages. For example, systemic knockout of signal transducer and activator of transcription 3 (*Stat3*) leads to embryonic lethality on embryonic day 6.5 [25]. Additionally, it is difficult to explore the specific function of a gene when it plays multiple roles in different cell types [26]. To overcome these limitations, researchers have developed tissue-specific knockout models using Cre/loxP recombination systems, which we introduce in the following sections.

### 2.1. Tissue-Specific Gene Ablation

**Figure 1:**
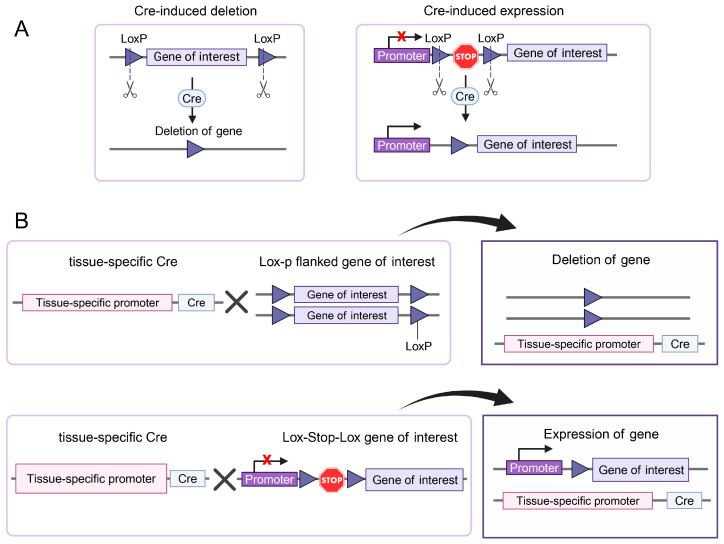
Cre-LoxP system for gene deletion and conditional gene expression. (**A**) Cre-LoxP system for gene manipulation. (**B**) Tissue-specific Cre-mediated gene deletion and activation.

**Figure 2:**
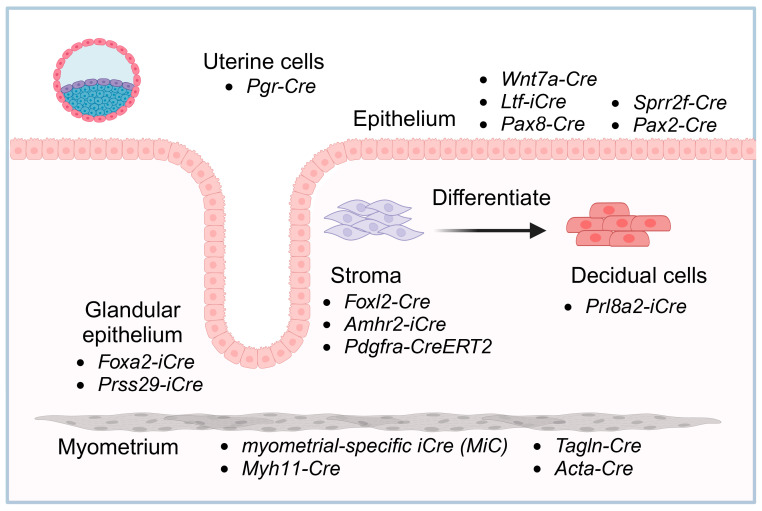
Schematic representation of uterine cell types and their specific Cre-driver lines. The diagram illustrates the major cellular components of the uterus, including the epithelium, stroma, decidua, and myometrium, along with the specific Cre-driver lines used for targeted genetic manipulation.

The uterus consists of two major compartments: the glandular endometrium and muscular myometrium. The endometrium can be further subdivided into subcompartments, including the luminal epithelium, glandular epithelium, and stroma. All compartments contain multiple cell types [27,28]. Understanding the molecular mechanisms regulating the interactions between these compartments cannot be accomplished using traditional germline manipulations. Sequence-specific recombinases have provided a groundbreaking solution to address the limitations of traditional germline knockout models, allowing the targeted ablation of genes in a tissue- and cell-specific manner. One of the most commonly used recombinases is Cre (Cause Recombination), which is derived from the bacteriophage P1. Cre recombination causes the excision of DNA sequences flanked by loxP (locus of Crossover in P1) sequences in a directional manner, where the orientation of loxP sites determines whether recombination results in deletion, inversion, or translocation of the intervening DNA segment (Figure 1A). Using homologous recombination, or more recently, CRISPR/Cas9 technology, researchers can insert loxP sequences into the mouse genome surrounding the sequences to be edited. These “floxed” genes (flanked with loxP sites) can be deleted in a tissue-specific manner using Cre recombinase expressed under the control of tissue-specific promoters [29]. To achieve precise genetic modifications in the Cre-Lox system, a strain carrying the Cre recombinase gene is crossed with a strain containing the gene of interest flanked by LoxP sites (floxed or Lox-Stop-Lox sites) (Figure 1B). This system allows for conditional, tissue- and/or cell-specific gene deletion or activation. This application has been used to induce recombination in multiple compartments of the female reproductive tract. Cre expression models have been developed that specifically cause recombination in these compartments and cell types. In this review, we present several uterine cell type-specific mouse models based on the Cre/loxP system that have been widely used to study uterine biology (Figure 2 and Table 1).

### 2.2. Uterine Cell-Specific Pgr-Cre Mouse Strain

Recombination in the female reproductive tract has lagged behind other systems due to the lack of a uterine-specific promoter to target recombinase transgenes to the uterus. This limitation was overcome by inserting Cre recombinase into loci with a major site of expression in the reproductive tract. The initial locus using this approach was *Pgr*, which is crucial for endometrial development and function. *Pgr* expression is limited to the reproductive axis, which consists of the pituitary gland, preovulatory granulosa cells, oviduct, uterus, and mammary glands. Insertion of the β-galactosidase gene (*Lacz*) into the *Pgr* locus demonstrated the limited targeting of transgenes to these tissues [48]. The *Pgr-Cre* knock-in mouse model was developed in 2005, in which Cre recombinase was inserted into the *Pgr* locus. Given that *Pgr* is a critical regulator of female reproduction [49], this model can only be used in mice heterozygous for the Cre allele. Mice heterozygous for Cre knock-in insertion exhibit normal fertility and are phenotypically similar to wild-type mice. When crossed with the *Rosa26R* reporter strain, which contains a lacZ or other reporter gene (GFP or Tomato) inserted into the Rosa26 locus, conditional expression is enabled using Cre recombinase. Cre recombinase activity is specifically detected in *Pgr*-expressing cells within the uterus, ovary, oviduct, pituitary gland, and mammary glands, similar to that in the *Pgr-Lacz* model [30]. Given this limited expression in reproductive tissues, Cre recombinase was inserted into the *Pgr* locus, making it an ideal tool for targeting gene manipulation in uterine cells, as long as it is determined that there is no impact of the ablation on pituitary or ovarian function. An alternative *Pgr* mouse model, *Pgr-BAC-iCre*, was generated by combining bacterial artificial chromosome (BAC) recombineering with transgenesis. The improved Cre (iCre) recombinase was inserted in-frame and placed under the control of the *Pgr* promoter within the BAC transgene. When crossed with *Rosa26R* reporter mice, the *PR-BAC-iCre* transgene expressed active iCre exclusively in *Pgr*-expressing cell lineages [31]. *Pgr-ires-Cre* was generated by inserting the Cre recombinase gene immediately downstream of the endogenous Pgr gene via an internal ribosome entry site (IRES), effectively directing Cre-mediated recombination in the female reproductive tract, particularly the endometrium [32]. Conditional knockout *Stat3* and *Gp130* using this strain exhibited the same embryo implantation defects as those observed in *Pgr-Cre* mice, indicating that *Pgr-iresCre* is a valuable tool for uterine-specific genetic modifications [32,50].

Researchers using *Pgr-Cre* mice have identified critical genes essential for successful implantation, such as Indian hedgehog (*Ihh*), muscle segment homeobox gene (*Msx*) family members 1/2 (*Msx1/2*), wingless-related MMTV integration site 4 (*Wnt4*), β-catenin (*Ctnnb1*), *Stat3*, vertebrate regulator of planar cell polarity Van Gogh-like 1/2 (*Vangl1/2*), and ERBB receptor feedback inhibitor 1 (*Erbbfi1*) [50,51,52,53,54,55,56]. The *Pgr-Cre* model has also revealed several key genes involved in decidualization. For example, conditional deletion of *Bone morphogenetic protein 2 (Bmp2)* restrains the decidual response, indicating its crucial role in decidualization [57]. Similarly, *Dicer*, an enzyme involved in microRNA processing, is essential for proper decidualization, as its deletion leads to enhanced stromal apoptosis and impaired proliferation in response to progesterone [58]. Chicken ovalbumin upstream promoter-transcription factor II (*COUP-TFII*) is a nuclear receptor prominently expressed in uterine stromal cells and is regulated by the progesterone-induced IHH signaling pathway from the epithelium. A study using the *Pgr-Cre* strain to conditionally ablate *COUP-TFII* in the uterus showed that the loss of *COUP-TFII* results in implantation failure and a decidualization defect. The lack of *COUP-TFII* results in increased estrogen activity in the epithelium, which hinders uterine receptivity during embryo implantation. Furthermore, *COUP-TFII* plays a critical role in regulating BMP2, which is essential for decidualization [59].

Studies using *Pgr-Cre* mice have provided considerable insights into the molecular mechanisms underlying embryonic implantation and decidualization. However, *Pgr* is also expressed in ovarian tissues, including the corpus luteum, which is a primary source of progesterone. This widespread expression can lead to unintended gene deletions in non-uterine tissues. For example, conditional deletion of *Lgr5* using *Pgr-Cre* resulted in infertility due to gene deletion in ovarian tissues rather than in the uterus [60]. Furthermore, Cre recombinase activity begins during the postnatal stage, which complicates the analysis of genes critical for normal uterine development and restricts the ability to examine their specific roles in the later stages of uterine function [61]. Forkhead box A2 (*Foxa2*) is a transcription factor mainly expressed in the uterine glands. *Foxa2* knockout mice using the *Pgr-Cre* model were completely aglandular, indicating that it is essential for the differentiation and development of glands. However, the infertility observed in adult mice was due to the absence of LIF, which is secreted by the glands, making it difficult to explore the role of *Foxa2* in pregnancy [62]. To address these limitations and achieve more precise uterine-specific gene targeting, researchers have developed alternative Cre mouse models. In the following section, we discuss Cre models designed to study distinct uterine compartments, including the epithelium, stroma, myometrium, and the process of decidualization.

### 2.3. Uterine Epithelium

Among the many Cre lines developed, *Wnt7a-Cre* and *Lactoferrin*-*iCre* (*Ltf-iCre)* mouse models have become invaluable tools for investigating uterine biology [33,34]. These models enable tissue-specific gene deletions in uterine epithelial cells, providing critical insights into the molecular mechanisms underlying uterine receptivity and implantation.

#### 2.3.1. *Wnt7a-Cre*

*Wnt7a* is expressed in the uterine epithelium during the fetal and neonatal stages [63]. Conditional deletion of *Wnt7a* using *Pgr-Cre* mice disrupts postnatal uterine gland morphogenesis and compromises adult fertility, revealing its pivotal role in the early development of the uterine epithelium [64]. The *Wnt7a-Cre* mouse line is active in the Müllerian duct epithelium during embryonic development, making it an invaluable tool for investigating uterine epithelial development and function [33]. A Müllerian duct-specific knockout study using *Wnt7a-Cre* mice revealed that LIM homeobox 1 (*Lhx1*) is essential for maintaining ductal progenitor cells for Müllerian duct elongation during female reproductive tract development [65]. Research using this Cre line revealed that uterine epithelial ESR1 is dispensable for estrogen-induced proliferation but essential for preventing apoptosis and regulating functions like lactoferrin (*Ltf*) production, ensuring a complete epithelial response during pregnancy [33]. While the *Wnt7a-Cre* model has proven highly effective for uterine studies, its Cre activity is not strictly limited to the uterine epithelium. Cre recombinase activity has also been detected in the oviduct epithelium, ovarian germ cells, hair follicular epithelium, and Pachytene spermatocytes [34,66]. These off-target effects require careful consideration, as they may complicate the interpretation of the results, especially in studies in which tissue specificity is critical.

#### 2.3.2. *Ltf-iCre*

*Ltf* expression is restricted to the post-pubertal stage and localized to uterine epithelial cells. This stage-specific expression allows for studies focusing on post-pubertal uterine function. *Ltf-iCre* knock-in mice express a codon-optimized iCre recombinase under the direction of the *Ltf* gene. The Cre recommendation is specifically for adult uterine epithelium and immature epithelium following estrogen treatment. Although the expression of these genes is regulated by estrogen, which can confound investigations of the impact of hormonal signaling, especially in cancer models, the ability to limit the activity to the adult mouse makes this model an essential tool for investigating uterine epithelial cell function in adult mice [34]. Research using the *Ltf-iCre* mouse model has uncovered several critical insights into uterine biology. Ablation of Lif receptor (*Lifr*) in the adult epithelium using this model revealed infertility due to impaired uterine receptivity, not embryo competency. Loss of *Lifr* disrupted STAT3 nuclear translocation and reduced the expression of the LIF-regulated gene *Msx1*, indicating its critical role in activating the LIF-STAT3 pathway for implantation success [67]. *Gp130* and *Stat3* are essential for uterine receptivity and implantation, as their conditional deletion using *Ltf-iCre* mice leads to implantation failure associated with increased uterine estrogenic responses [50]. Furthermore, while conditional knockout of *Foxa2* using *Pgr-Cre* mice lacks glands, conditional deletion of *Foxa2* in the adult mouse uterus using the *Ltf-iCre* model exhibited normal uterine morphology but was completely infertile due to the absence of LIF [68]. The *Ltf-iCre* mouse model has also demonstrated the role of *Vangl2*-mediated crypt formation and cell polarity of the luminal epithelium, which are essential for successful embryo implantation [69]. Together, these studies show that the *Ltf-iCre* model is a crucial tool for understanding how the adult uterine epithelial structure and function contribute to pregnancy success.

#### 2.3.3. Uterine Glandular Epithelium-Specific Strains

FOXA2 is a transcription factor expressed exclusively in the glandular epithelial cells of the mouse and human endometrium, playing a dual role in regulating gland development during the neonatal period and gland function in adulthood [70,71]. Investigation using the *Foxa2-Cre* mouse model has showed that the ESR1 is crucial for regulating uterine epithelial lineage specification and maintaining homeostasis [72]. However, Cre recombinase activity driven by the *Foxa2* promoter is not limited to the uterine glandular epithelium; it extends to other tissues, such as the node, notochord, floorplate, and various endoderm-derived organs, including the liver, lung, pancreas, and gastrointestinal tract [35]. Extensive Cre activity across multiple tissues can lead to unintended gene deletions outside the uterus. Therefore, it may not be ideal for studies exclusively targeting the uterine glandular epithelium.

The *Prss29-Cre* mouse line provides a new tool for studying uterine gland function during pregnancy by specifically targeting the uterine glandular epithelium. Both serine protease 29 (*Prss29*) mRNA and Cre activity are limited exclusively to the uterine glandular epithelium following implantation, with no detection in other regions of the female reproductive tract. Knockout of *Foxa2* in post-implantation uterine glands using this Cre model led to complete infertility after the first pregnancy, revealing the critical role of *Foxa2* in gland function and fertility [36]. This model offers a valuable resource for investigating the specific contributions of uterine glands to pregnancy.

#### 2.3.4. Other Epithelium-Specific Cre Lines

Several other Cre lines have been used to delete genes in the uterine epithelium, each with distinct characteristics and applications. *Pax8* is a transcription factor expressed in the Müllerian epithelium of the uterus and oviducts but not in the ovaries. Cells expressing *Pax8* are crucial for maintaining the endometrial epithelium and are considered potential cells of origin for serous endometrial carcinoma [73]. The *Pax8*-Cre model was developed to study uterine and oviduct functions [37]. Researchers have utilized *Pax8*-Cre mice to model the initiation, progression, and invasion of uterine endometrioid carcinomas. Deletion of the *Arid1a* gene in the endometrial epithelium using this model have shown that its loss promotes invasion and metastasis [74]. Additionally, *Pax8*-Cre mice with heterozygous loss of *Pten* in the tubal epithelium develop hyperplasia, while homozygous loss leads to cancer in the uterus and oviducts [75]. The *Sprr2f*-Cre transgene has also been developed to target genes within endometrial epithelium. However, Cre activity is also observed in the cerebellum and kidney [38].

Transgenic mouse models with epithelial-specific Cre lines have elucidated the roles of various genes in uterine development, gland formation, and reproductive processes. Deletion of *Esr1* using *Pgr-Cre* resulted in a complete block of gland development, while *Pax2-Cre*-mediated deletion in the embryonic Müllerian duct epithelium led to unbranched glands that failed to express glandular LIF, disrupting implantation chamber formation and embryo alignment. Surprisingly, glands in *Ltf-iCre*; *Esr1-floxed* mice were branched and exhibited reduced LIF expression, resulting in delayed implantation but normal pup delivery. Therefore, branched glands are necessary for glandular LIF secretion and implantation success [76].

### 2.4. Stroma

Studying uterine stromal cells remains challenging due to the lack of uterine stroma-specific Cre mouse models. Anti-Müllerian hormone receptor type 2 (*Amhr2*) is expressed in mesenchyme adjacent to the Müllerian ducts, as well as in Sertoli, Leydig, and granulosa cells [77]. The *Amhr2-IRES-Cre(Bhr)* mouse strain has been employed for performing genetic recombination in these cells [39]. By crossing with reporter strains, researchers have observed that *Amhr2*-driven Cre recombinase activity is predominantly confined to the Müllerian duct mesenchyme-derived stroma and myometrium in uterus [78]. Over the past 20 years, this *Amhr2-IRES-Cre(Bhr)* mouse model has been widely used, especially to ablate genes in the reproductive tract including ovary, uterus, and testes. Conditional deletion of the Progesterone receptor membrane component 1 (*Pgrmc1*) gene using *Amhr2-Cre* led to subfertility and the development of endometrial cysts in female mice, revealing its significance in maintaining uterine tissue homeostasis and fertility [79]. Similarly, targeted ablation of *Stat3* in ovarian granulosa and uterine stromal cells via *Amhr2-Cre* resulted in impaired fertility due to compromised embryo implantation and placental development [80]. However, this model has exhibited variability in recombination efficiency and unintended genetic modifications in non-target tissues [81]. To address these issues, researchers developed the *Amhr2-iCre(Fjd)* model by inserting a codon-optimized iCre into exon 1 of the *Amhr2* gene. *Amhr2-iCre(Fjd)* mediates consistent genetic recombination across uterine epithelial, stromal, and myometrial cells, whereas the *Amhr2-IRES-Cre(Bhr)* model displayed inter-mouse variability in uterine cell recombination. Notably, both *Amhr2-iCre(Fjd)* and *Amhr2-IRES-Cre(Bhr)* exhibit Cre activity as early as the four-cell embryo stage, leading to global genetic modifications [82]. This early embryonic activity suggests that these models may not be suitable for studies requiring stroma-specific gene ablation post-development in uterus, as unintended widespread recombination could confound results.

Forkhead box L2 (*Foxl2*) is a transcription factor crucial for follicular maturation and maintenance of ovarian identity. Several *Foxl2-Cre* mouse models have been developed to facilitate targeted gene manipulation. In *Foxl2-Cre* model, a Cre recombinase sequence replaces the translational start site of the *Foxl2* gene, enabling Cre expression under the control of the endogenous *Foxl2* promoter [40]. Using *Foxl2-Cre*; *R26R-LacZ* mouse model, it has been demonstrated that *Foxl2* is expressed not only in the ovary but also in other components of the female reproductive tract, including the uterus, cervix, and oviduct. In the uterus, *Foxl2* expression is first observed in the neonatal mesenchyme and, during uterine maturation, persists in the stroma and the deep inner myometrial layer (IML). In adults, *Foxl2* is expressed in the differentiated stromal layer but is no longer present in the myometrium [83]. The *Foxl2-CreERT2* inducible model incorporates a CreERT2 cassette downstream of the Foxl2 gene’s stop codon. CreERT2 is a tamoxifen-inducible Cre recombinase, and administration of tamoxifen activates Cre recombinase in *Foxl2*-expressing cells, allowing for precise temporal regulation of gene manipulation [84]. *Foxl2-IRES-CreERT2* model features an IRES-CreERT2 sequence inserted at the *Foxl2* gene’s stop codon, enabling inducible Cre recombinase expression in *Foxl2*-expressing tissues [41,85]. It is important to note that while *Foxl2-Cre* models are effective for targeting genes in the uterine stroma, they are also expressed in ovarian granulosa cells. Therefore, when using these models to study uterine-specific functions, researchers should consider the potential impact on ovarian tissues as well as the direct effects of tamoxifen administration on the uterus. Tamoxifen can function as a weak estrogen agonist in the endometrium, potentially leading to endometrial proliferation and other uterine effects [86,87].

Platelet-derived growth factor receptor alpha (*Pdgfrα*) is a cell surface receptor protein that plays a crucial role in cell proliferation, migration, and survival. Using *Pdgfrα-EGFP* mouse model, PDGFRα has been found to be expressed throughout the reproductive tract in distinct regions. In the uterus, PDGFRα+ cells are densely distributed throughout the myometrium and stroma [88]. The *Pdgfrα*-*CreERT2* knock-in mouse model utilizes tamoxifen-inducible Cre recombinase (CreERT2) driven by the *Pdgfrα* promoter [42]. By crossing with *Rosa26*-tdTomato reporter mouse, Tomato expression is specifically observed throughout the endometrial stromal compartment. Importantly, no expression is detected in the myometrium or luminal/glandular epithelium, indicating the model’s specificity for stromal cells [89]. Another study has applied this model to study the Cre recombination in the placenta. *Pdgfrα-CreER*; *Rosa*-mT/mG mice were administered tamoxifen via oral gavage at different gestational stages. mEGFP expression was detected in the junctional zone (JZ) and chorionic plate (CP) of the placenta. Importantly, a single dose of tamoxifen was sufficient to induce recombination, indicating the efficacy of this system for targeted Cre expression in the placenta [90].

In summary, the development of advanced Cre mouse models, including *Amhr2-Cre*, *Foxl2-Cre*, and *Pdgfrα-CreERT2*, has significantly advanced our ability to study uterine stromal cells. Each model offers unique advantages and limitations, with varying specificity and recombination efficiencies in different uterine compartments.

### 2.5. Decidual Cells

The *Pgr-Cre* mouse model has been widely used to study the role of genes in decidualization. However, its utility is limited to genes that are not essential for implantation or ovarian function, as the knockout of critical implantation-related genes leads to implantation failure, preventing the study of their specific roles in decidualization. This limitation has hindered a comprehensive understanding of the genetic and molecular mechanisms underlying decidualization for some critical genes. To overcome these challenges, researchers have developed alternative Cre mouse models with enhanced specificity for decidual cells. One such model is the *Prl8a2-iCre* mouse, which expresses an iCre under the control of the endogenous prolactin family 8, subfamily a, member 2 (*Prl8a2*) promoter. *Prl8a2* is specifically expressed in decidual cells following embryo implantation, making this model particularly valuable for studying genes involved in decidualization without affecting the implantation process. In this model, iCre activity initiates around GD 5.5 in the primary decidual zone and persists into mid-gestation, with expression concentrated in the anti-mesometrial region. Importantly, the use of the Sun1-GFP reporter strain has confirmed that iCre activity is absent in other reproductive tissues, such as the ovary, oviduct, and pituitary gland [43]. This decidua-specific Cre model enables researchers to investigate the functions of genetic networks and cellular dynamics associated with decidualization and infertility.

### 2.6. Myometrium

Several Cre mouse models have been used to study the molecular and physiological mechanisms of the uterine myometrium. The *Tagln-Cre* model uses the transgelin (*Tagln*) promoter to drive Cre recombinase expression in smooth muscle cells, with Cre mRNA expression being highest in the aorta, intestine, and uterus [44]. This model is highly specific because no Cre activity has been observed in non-smooth muscle cells. Similarly, the *Acta2-CreERT2* model employs the alpha-smooth muscle actin *(Acta)* promoter to enable tamoxifen-inducible Cre activity, allowing for temporal control over gene recombination [45]. However, heterogeneous staining was observed in the uterus, suggesting that Cre is not active in all uterine smooth muscle cells [91]. The *Myh11-Cre* model has been used to investigate the role of PGR in myometrial function and adaptation during pregnancy [46]. *Pgr* ablation in smooth muscle cells leads to subfertility, oviductal embryo retention, and impaired myometrial remodeling. These mutant uteri exhibit a reduction in oxytocin-stimulated contractility, altered expression of calcium homeostasis genes, including phosphodiesterase type 5A (*Pde5a*) and phospholipase C Beta 4 (*Plcb4*), discontinuous myofibers, and a disarrayed extracellular matrix at mid-pregnancy [92]. Recently, a myometrial-specific Cre recombinase transgenic mouse strain (Myometrial-specific M-iCre, MiC) was generated using the rat calbindin-D9K (CaBP9K) promoter, which drives transgene expression specifically in the myometrium. By crossing with Rosa-mT/mG reporter mice, EGFP fluorescence was observed only in the myometrium, with no EGFP expression detected in extrauterine tissues, including the smooth muscle of the heart, intestine, and kidney [47]. Together, these Cre models provide valuable tools for studying myometrial function during pregnancy.

## 3. Endometrial Organoids

**Figure 3:**
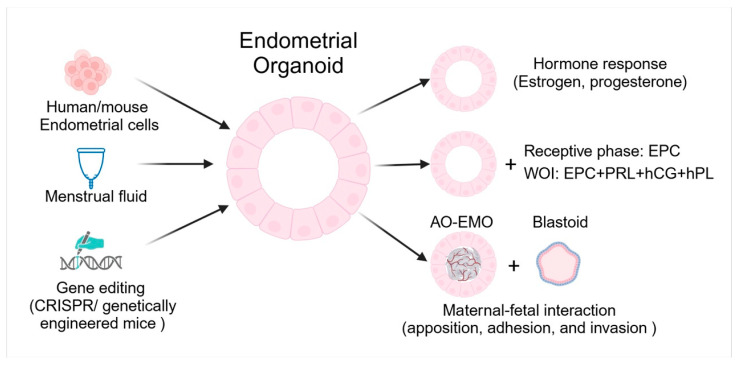
Endometrial organoid model for studying uterine function and maternal-fetal interactions. The development and application of endometrial organoids derived from diverse sources and their utility in modeling hormone responses, the receptive phase, and maternal-fetal interactions are graphically summarized.

Endometrial organoids derived from primary endometrial cells of humans and mice have become pivotal in studying the molecular mechanisms of uterine biology. Endometrial cells isolated from human endometrial biopsies or mouse uteri are embedded in a Matrigel matrix and cultured in specialized media containing growth factors and Wnt ligands. These cells self-organize into spheroid or gland-like structures that closely replicate the cellular architecture and function of the native endometrium. They exhibit long-term expansion capabilities and respond to hormonal cues, such as estrogen and progesterone, mimicking the cyclical changes in the endometrium [93]. Endometrial organoids can also be generated from menstrual fluid [94]. Menstrual fluid-derived organoids provide a non-invasive alternative to endometrial biopsies. Furthermore, these organoids respond to hormones in a manner similar to that of native endometrial tissue [94,95]. Here, we discuss the application of endometrial organoids in studying endometrial changes in response to hormones, the implantation process, and embryo-maternal interactions, providing a controlled model to investigate how the endometrium supports early pregnancy (Figure 3).

### 3.1. Respond to Hormones

Human endometrial organoids have a remarkable ability to respond to hormonal signals, particularly estrogen and progesterone, effectively mimicking the changes observed in the uterine lining throughout the menstrual cycle. When exposed to estrogen, organoids exhibit characteristics of the proliferative phase, including enhanced cell proliferation and glandular development. In contrast, treatment with progesterone induces a transition to the secretory phase phenotype, marked by increased glandular folding and the production of secretory proteins [93,96]. This hormone responsiveness enables endometrial organoids to replicate the natural fluctuations of the menstrual cycle, providing a robust platform for investigating endometrial physiology and related disorders.

### 3.2. Implantation

Human endometrial receptivity plays a pivotal role in determining pregnancy success; however, in vivo studies are limited by ethical restrictions. Endometrial organoids derived from biopsies of women with primary infertility and normal fertility were treated with estradiol (E2), medroxyprogesterone acetate (MPA), and cyclic adenosine monophosphate (cAMP) to model the receptive phase of the endometrial epithelium. Proteomic analysis of apical secretions identified dysregulated proteins between the fertile and infertile groups. Notably, trophoblast progenitor spheroids treated with secretions from organoids derived from infertile individuals exhibited significantly compromised adhesion to epithelial cell monolayers compared to those treated with secretions from organoids derived from fertile donors. This suggests that dysregulation of endometrial epithelial secretions may impair trophoblast adhesion, potentially contributing to implantation failure in infertility [97]. Researchers have established a WOI endometrial organoid system by supplementing the culture system with prolactin (PRL), human chorionic gonadotropin (hCG), human placental lactogen (hPL), E2, MPA, and cAMP. These WOI organoids effectively mimic the receptive endometrium by replicating its structural and cellular complexity. They exhibit key implantation features, such as hormone responsiveness, decidualization, extracellular matrix remodeling, pinopode formation, and cilia generation, modeling critical processes like the proliferation-to-secretory transformation and epithelial-mesenchymal transition [98].

### 3.3. Maternal-Fetal Interactions

The blastoid model, which mimics the structure and development of natural blastocysts, has been generated using naïve human pluripotent stem cells (PSC) under specific conditions. It accurately replicates the three key embryonic lineages: the trophectoderm (TE), which contributes to the placenta; epiblast (EPI), which forms the embryo; and primitive endoderm (PrE), which supports early development [99]. The blastoid attach to the hormonally stimulated endometrium through polar TE (pTE), which is defined by its contact with EPI, similar to human blastocytes [99,100]. However, it cannot attach to the unstimulated endometrial cells. STAT signaling, driven by IL-6 and its receptors in the EPI, is crucial for blastoid development. LIF enhances blastoid formation, while inhibition of GP130 or Hippo kinases (MST1/2) leads to trophospheres that cannot attach. EPI signals are essential for pTE maturation and enabling attachment. Transcriptome analysis suggests the molecular mediators of blastocyst-endometrium interactions. A polar-like TE state, regulated by EPI, governs the implantation timing [100].

The development of endometrial assembloids comprising hormone-responsive apical-out endometrial organoids (AO-EMO) and human embryonic stem cell-derived blastoids has allowed for the study of the embryo-endometrial interface in vitro [101,102,103]. The AO-EMO model closely mimics the architecture of the endometrium, with dense stromal cells and a self-organized endothelial network surrounding the apical surface epithelium. When cocultured with blastoids, the system effectively recapitulated the key stages of implantation, including apposition, adhesion, and invasion [101]. The epithelium directs polarized and proper adhesion, and its absence leads to disordered blastoid adhesion. Stromal cells further support adhesion and invasion by modulating the epithelial barrier. Syncytial differentiation at the trophoblast-endometrium interface is a key factor in breaching the epithelial barrier and promoting stromal invasion, with the degree of syncytial differentiation potentially influencing the site of trophoblast adhesion and overall implantation success. These findings emphasize the critical roles of the endometrial epithelium and stromal cells in implantation, with syncytial differentiation emerging as a key determinant of successful adhesion and invasion [103].

### 3.4. Genetic Manipulation

A significant advantage of endometrial organoids is their amenability to genetic manipulation. Techniques like CRISPR/Cas9-mediated gene editing and viral vector-based transduction have been employed to introduce specific genetic modifications, allowing researchers to investigate the roles of individual genes in endometrial function and implantation processes [104]. However, the efficiency of CRISPR/Cas9 is not absolute; some cells may not undergo the intended genomic edits, and there is a potential risk of off-target effects [105]. To address these challenges, researchers can utilize endometrial cells derived from genetically engineered mice to study the functions of specific genes. Using *Axin2-rtTA;tetO-H2BJGFP* mice, FACS-sorted *Axin2*+ cells showed significantly higher organoid-forming efficiency than *Axin2*− cells, which rarely formed organoids and failed to survive beyond a few passages. *Axin2*+-derived organoids resembled the in situ endometrium, expressing markers of ciliated, glandular, and epithelial cells, while *Axin2*− organoids showed poor growth and non-responsiveness to WNT signaling. Mutant organoids from *Axin2-rtTA;tetO-Cre;R26-Pik3ca; Ctnnb1fl(ex3)/+* endometrial cells exhibited both WNT signaling activation (driven by the *Ctnnb1fl(ex3)/+* allele, which stabilizes *Ctnnb1* in *Axin2*-expressing cells) and oncogenic Pik3ca expression (controlled by the *R26-Pik3ca* allele in the same *Axin2*-expressing cells). These organoids exhibited higher formation efficiency and included larger, irregular, and branched tumoroid structures compared with the controls [106]. Endometrial organoids derived from *Esr1* null epithelial cells (*Esr1* null organoids) developed a multilayered stratified squamous epithelium with basal cells, while wild-type (WT) organoids formed a single layer of columnar epithelium. Interestingly, co-culturing *Esr1* null organoids with WT uterine stromal cells inhibited basal cell development. Additionally, estrogen treatment in both organoid-stromal co-cultures and *Pgr-Cre Esr1* conditional knockout mice enhanced the expression of basal epithelial cell markers. These findings indicate that ESR1 regulates uterine epithelial lineage plasticity and homeostasis, with ESR1-mediated paracrine factors from the stroma playing a pivotal role in maintaining the luminal-to-basal differentiation balance [72]. Therefore, endometrial organoids serve as versatile tools for genetic manipulation, enabling the investigation of gene-specific roles in uterine function.

## 4. Omics Technologies

**Figure 4:**
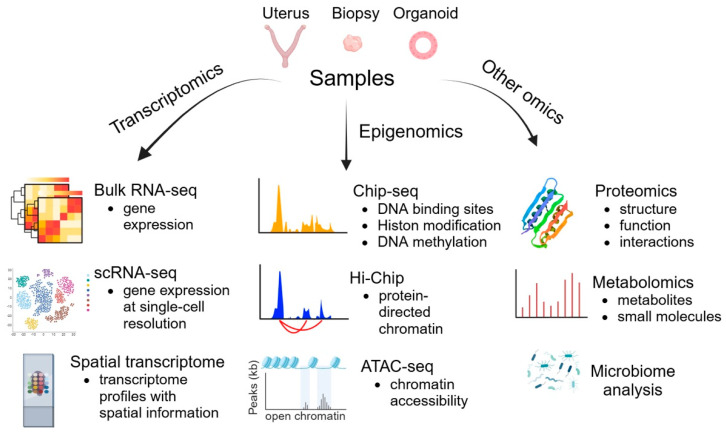
Multi-omics approaches in uterine research. Various omics approaches, including transcriptomics and epigenomics, are illustrated along with the types of data they generate.

Omics provides a powerful framework for understanding the complex molecular and cellular mechanisms underlying implantation, offering insights into gene expression, epigenetic modifications, and signaling pathways [107]. Here are the key aspects in which omics technologies have advanced our understanding of implantation (Figure 4):

### 4.1. Transcriptomics

Bulk RNA sequencing (RNA-seq) has become the gold standard for profiling gene expression. By comparing receptive and non-receptive endometrium or successful and failed implantation, bulk RNA-seq can identify differentially expressed genes, such as those involved in endometrial receptivity and trophoblast invasion [108]. Moreover, it facilitates the discovery of novel non-coding RNAs and alternative splicing events that influence implantation [109]. Bulk RNA-seq measures the average gene expression across a heterogeneous population of cells, which can obscure the contributions of specific cell types [110]. As the uterus consists of various cell types (e.g., epithelial, stromal, and immune cells), Bulk RNA-seq is unable to discern gene expression changes unique to specific cell populations and capture spatial dynamics during implantation, decidualization, and placentation, which are critical for understanding the underlying molecular mechanisms. To overcome the limitations of bulk RNA-seq, researchers have increasingly used single-cell RNA sequencing (scRNA-seq) and spatial transcriptomics, which have revolutionized our understanding of implantation at both cellular and spatial levels [27,111,112].

#### 4.1.1. scRNA-seq

The human endometrium exhibits a complex and dynamic cellular composition that is poorly understood. scRNA-seq enables the analysis of gene expression at single-cell resolution, revealing cellular heterogeneity and identifying specific cell populations within the endometrium [113]. Several studies using scRNA-seq have provided important insights into the cellular composition of the human endometrium [114,115,116]. Since the endometrium exhibits significant heterogeneity both between individuals and within the same individual, a large sample size is required to reflect dynamic changes over time (throughout the menstrual cycle) and across various tissue microenvironments. A recent study created a single-cell reference atlas (termed HECA) by integrating six published scRNA-seq datasets with their newly generated single-cell seq dataset. HECA resolves long-standing challenges by establishing consensus definitions for endometrial cell types and by identifying previously unreported populations. The functional layer reveals intricate stromal-epithelial interactions mediated by transforming growth factor beta (TGFβ) signaling. In the basalis layer, signaling between fibroblasts and epithelial populations expressing progenitor markers is crucial for endometrial regeneration [27].

In humans, studying implantation directly in vivo is not feasible due to ethical and practical limitations. In contrast, mouse models allow researchers to study implantation across well-defined time points, from the pre-receptive stage to post-implantation stage [117]. Using scRNA-seq to compare mouse uteri on gestation day (GD) 2.5 (pre-receptive stage) and GD 3.5 (receptive stage), researchers identified global gene expression changes in the luminal and glandular epithelium associated with uterine receptivity. In the luminal epithelium, the differentially expressed genes (DEGs) were significantly enriched in processes like cell death, proliferation, and protein metabolism, and hub genes included polyadenylate-binding protein 1 (*Pabpc1*), E1A Binding Protein P300 (*Ep300*), and *Stat3*. In the glandular epithelium, the DEGs were enriched in transport and protein metabolism, revealing the critical pathways driving uterine receptivity [118]. Using scRNA-seq on implantation sites (IS) and inter-implantation sites (IIS) in GD 4.5 mouse uteri, a transcriptomic atlas was generated to uncover global gene expression changes associated with implantation across different cell types. The overall proportions of most cell types were unchanged, except for lymphatic endothelial cells (LECs) and B cells. Notably, the proportion of proliferating LECs (LECp) significantly decreased at IS, while proliferating stromal and vascular endothelial cells significantly increased, indicating dynamic cellular changes during implantation [119,120]. Using published E4.5 mouse blastocyst scRNA-seq data, predicted ligand–receptor interactions between uterine LE and mural trophectoderm (mTE) revealed several key pathways, including Ras, Ras-associated protein 1 (Rap1), phosphatidylinositol 3-kinase (PI3K)/protein kinase B (AKT), mitogen-activated protein kinase (MAPK), and WNT signaling, as well as cytokine interactions and actin regulation, revealing their roles in implantation [119]. By combining scRNA-seq and mouse models, researchers can dissect the cellular and molecular dynamics of implantation with unprecedented resolution.

#### 4.1.2. Spatial Transcriptomics

Spatial transcriptomics enables the visualization of gene expression patterns in their native locations, providing insights into the tissue architecture and cellular interactions. However, due to the limited single-cell resolution of many spatial methods, integration with single-cell or single-nucleus transcriptomes is necessary to accurately distinguish individual cell types and subpopulations [121]. By integrating single-cell, single-nucleus, and spatial transcriptional profiling, analyses of the cellular states and spatial organization of human endometrial cells during the proliferative and secretory phases of the menstrual cycle in women of reproductive age revealed that WNT and NOTCH signaling are key drivers of ciliated and secretory epithelial differentiation [111]. A study integrating scRNA-seq data of the WOI endometrium with published spatial transcriptomic data of the secretory endometrium identified significant time-associated molecules from stromal and epithelial cells that interact with immune cells. Notable interactions included stromal-immune pairs (CXCL12-CXCR3/4, ANXA1-FPR1/3, HLA-C-FAM3C, C3-ITGB2/ITGAM, CD55-ADGRE5) and epithelial-immune pairs (TGFB2-TGFBR1/2, SPP1-CD44, ANXA1-FPR1/3, CDH1-ITGB1/ITGAE, CD55-ADGRE5), revealing their co-expression and roles in the WOI [122].

Spatial transcriptomics and scRNA-seq were used to analyze the transcriptome profiles with spatial information at the embryo implantation site (GD 7.5) in mice, revealing 11 distinct domains with unique gene signatures. These include the mesometrial and anti-mesometrial myometrium, mesometrial decidua enriched with natural killer cells, vascular sinus zone, fetal–maternal interface, primary, transition, and secondary decidual zones, undifferentiated stroma, uterine glands, and embryos. Single-cell RNA signatures clarified the cell-type composition within these regions. Functional annotations reveal distinct metabolic, immune, and cellular behaviors regulated by region-specific endocrine and paracrine signals, providing valuable insights into the molecular mechanisms underlying pregnancy [123]. To understand regional changes during early pregnancy, researchers have utilized spatial transcriptomics combined with scRNA-seq to create a detailed spatiotemporal atlas of mouse implantation sites during the decidualization process (GD 5.5–GD 9.5). Functional decidual hubs were identified, consisting of decidual stromal cells, immune cells, uterine endothelial cells, and trophoblasts, which displayed dynamic spatial and temporal changes in early pregnancy. The generated data are accessible through the Mouse Embryo Implantation Site Spatiotemporal Atlas (MEISSTA) interactive data portal (https://meissta.com/, accessed on 20 January 2025) [124]. Taken together, the integration of spatial transcriptomics with single-cell and single-nucleus transcriptomics provides a comprehensive understanding of the spatial and temporal dynamics of endometrial biology.

### 4.2. Epigenomics

Epigenomics studies the complete set of epigenetic modifications of the genetic material of a cell. Chromatin Immunoprecipitation Sequencing (ChIP-seq) is a powerful method for identifying genome-wide DNA-binding sites for transcription factors and other proteins. Epigenetic mechanisms, including histone post-translational modifications (PTMs), are critical regulators of transcriptional changes during the endometrial cycle [125,126]. Global levels of H3K9ac, H2A lysine 5 acetylation (H2AK5ac), H3K14ac, and H4K8ac increase during the early proliferative phase, decline during the late proliferative phase, and rise post-ovulation to support ESC decidualization and implantation. In the late secretory phase, acetylation levels decrease with reduced hormone levels [125,126,127]. While global histone methylation levels remain stable throughout the cycle [128], specific changes in histone methylation have been observed in particular genes. H3K27me3 enrichment at the *HOXA10* promoter is higher during the proliferative phase than during the secretory phase, aligning with the dynamic expression of *HOXA10*, which is downregulated in the proliferative phase and highly expressed in the secretory phase [129,130]. CUT and RUN is a more streamlined and cost-effective alternative to ChIP-seq than other methods. This technique offers several advantages over ChIP-seq, including a higher resolution, reduced background noise, and lower sample input requirements. By leveraging a targeted enzymatic cleavage approach, CUT and RUN eliminates the need for cross-linking and sonication, simplifying the workflow and minimizing potential artifacts associated with ChIP-seq [131].

Hi-ChIP is a protein-centric chromatin conformation method used to identify chromatin loops associated with protein-bound regions, which are vital for gene regulation [132]. By targeting chromatin interactions marked by H3K27Ac, a histone modification linked to active enhancers, this technique can enrich for loops that connect enhancers to their target promoters. This enables researchers to map functional genomic elements and understand their roles in regulating gene expression [133,134].

ATAC-seq is a cutting-edge technique used to study chromatin accessibility. Using ATAC-seq to map chromatin changes in undifferentiated and decidualizing human endometrial stromal cells, 185,084 open DNA loci were identified, revealing that changes in chromatin accessibility were associated with differential gene expression. Enriched transcription factor motifs, including a basic leucine zipper within a triple motif (with estrogen receptor and Pax domain sites), were found in Alu elements. While other transposable elements were less represented, some contributed to the regulatory landscape of decidualization [135]. Furthermore, single-cell ATAC-seq across the menstrual cycle identified unique chromatin accessibility patterns in epithelial and stromal cells, indicating that dynamic chromatin remodeling supports a receptive environment. Through an integrated analysis of enriched transcription factor binding sites in dynamic chromatin regions, ChIP-seq data, and gene expression profiles, the implantation window was found to coincide with the pervasive co-option of transposable elements into the regulatory chromatin landscape of decidualizing cells [136,137].

### 4.3. Other Omics

Other omics technologies, including proteomics, metabolomics, and microbiome analyses, have also contributed to our understanding of implantation. Proteomic analysis of endometrial tissues has provided insights into the signaling molecules and receptors that are essential for embryo implantation [138]. Using isobaric tag for relative and absolute quantitation (iTRAQ)-based proteomics, researchers identified 263 differentially expressed proteins (DEPs), which are involved in protein translation, mitochondrial activity, oxidoreductase function, and fatty acid and amino acid metabolism, and are associated with recurrent implantation failure (RIF) in women. Among them, tubulin polymerization-promoting protein family member 3 (TPPP3), S100 calcium-binding protein A13 (S100A13), 17β-hydroxysteroid dehydrogenase 2 (HSD17B2), and alpha-2-glycoprotein 1, zinc binding (AZGP1) were identified as potential biomarkers for endometrial receptivity [139]. A recent review summarized the findings of proteomic analyses of the endometrium around the time of embryo implantation, offering valuable insights into endometrial receptivity in humans [140].

Metabolomics has also advanced our understanding of the role of the uterine environment in implantation. Metabolomic analysis of the endometrium during the receptive phase has identified 925 metabolites, predominantly lipids, with polyunsaturated fatty acids (PUFAs) being the most abundant. Notably, women with endometriosis and recurrent implantation failure exhibit reduced PUFA levels compared to those with male factors or unexplained infertility, suggesting that PUFA deficiency might impair endometrial function in these conditions [141]. Using a microfluidic system, murine embryos were cultured in endometrial epithelial cell-conditioned media (CM). The blastocyst formation rates were significantly increased in the CM group compared to the control. Metabolomic analysis revealed the upregulation of metabolites involved in arginine, proline, and pyrimidine metabolism, suggesting that endometrial epithelium secretions positively influence embryo development by modulating specific metabolic pathways [142].

The endometrial microbiome plays a significant role in embryo implantation and in reproductive outcomes. Microbiome analysis of endometrial fluid has shown that women with higher microbial diversity in the endometrium exhibit improved implantation success [143]. In patients with RIF, the microbiota profile of endometrial biopsies differed significantly from that of controls, with significantly lower *Lactobacillus* dominance and an association with genera such as *Prevotella*, *Streptococcus*, and *Dialister* [144].

## 5. Conclusions

Implantation is a highly complex and essential process for establishing a successful pregnancy, requiring intricate interplay between the receptive uterus and competent embryo. Advances in molecular biology, omics technologies, innovative in vivo Cre/loxP genetically engineered mouse models, and in vitro endometrial organoid models have significantly enhanced our understanding of the molecular and cellular mechanisms governing implantation and uterine biology. These cutting-edge approaches not only deepen our understanding of reproductive biology but also pave the way for novel strategies to address infertility, improve pregnancy outcomes, and tackle uteroplacental disorders.

## Figures and Tables

**Table 1:** Summary of the Cre mouse strains.

Tissue	Strain	Modifications	Advantages	Considered
Uterine	*Pgr-Cre* [30]	Knock-in	Targets uterine Pgr-expressing cells; widely used for uterine studies.	Also active in ovaries, pituitary, and mammary gland; may cause unintended gene deletions; activity begins postnatally, complicating early development studies.
	*Pgr-BAC-iCre* [31]	Transgenic	Enable to breeding of homozygous mice.	Similar off-target expression concerns as Pgr-Cre.
	*Pgr-IRES-Cre* [32]	Transgenic	Enable to breeding of homozygous mice.	Similar off-target expression concerns as Pgr-Cre.
Epithelium	*Wnt7a-Cre* [33]	Transgenic	Targets uterine epithelium from early stages; used for Müllerian duct studies.	Off-target activity in oviduct epithelium, ovarian germ cells, and other tissues.
	*Ltf-iCre* [34]	Knock-in	Restricts Cre activity to post-pubertal uterine epithelium; useful for adult uterine studies.	Expression is estrogen-dependent, complicating hormone-related studies.
	*Foxa2-Cre* [35]	Knock-in	Effective for studying glandular epithelium.	Off-target activity in multiple tissues (liver, pancreas, lung, etc.); limits uterine specificity.
	*Prss29-Cre* [36]	Knock-in	Targets uterine glandular epithelium exclusively after implantation.	Limited to glandular function post-implantation, restricting broader uterine studies.
	*Pax8-Cre* [37]	Knock-in	Targets Müllerian epithelium of the uterus and oviducts.	Potential off-target effects.
	*Sprr2f-Cre* [38]	Transgenic	Targets endometrial epithelium.	Cre activity detected in the cerebellum and kidney.
Stroma	*Amhr2-IRES-Cre(Bhr)* [39]	Transgenic	Commonly used for reproductive tract studies; targets both ovarian and uterine stromal cells.	Variability in recombination efficiency; off-target effects in non-uterine tissues; early embryonic Cre activity causes global recombination.
	*Foxl2-Cre* [40]	Knock-in	Useful for stroma-specific studies; allows early uterine gene deletion.	Active in ovary, which complicates uterine-specific studies.
	*Foxl2-CreERT2* [41]	Knock-in	Temporal control of gene recombination; Enables precise timing for gene deletion in stroma.	Active in ovary; Tamoxifen can have estrogenic effects on the endometrium, potentially confounding results.
	*Pdgfrα-CreERT2* [42]	Knock-in	Temporal control of gene recombination; high specificity for stromal cells.	Requires tamoxifen induction, which has estrogenic effects; not suitable for early-stage gene deletions.
Decidua	*Prl8a2-iCre* [43]	Knock-in	Targets decidual cells exclusively; avoids effects on implantation and ovarian function.	Limited to decidual cell studies; may not be useful for genes involved before GD 4.5.
Myometrium	*Tagln-Cre* [44]	Transgenic	High specificity for myometrial cells; no activity in non-smooth muscle cells.	Not specific to uterine smooth muscle cells.
	*Acta2-CreERT2* [45]	Transgenic	Temporal control of gene recombination.	Cre is not active in all uterine smooth muscle cells are targeted.
	*Myh11-Cre* [46]	Transgenic	Targets myometrial cells and enables study of labor mechanisms.	Not specific to uterine smooth muscle cells.
	*MiC (Myometrial-specific M-iCre)* [47]	Knock-in	Highly specific for uterine myometrium; avoids non-reproductive off-target effects.	NA

## Data Availability

Not applicable.

## References

[1] Massimiani M., Lacconi V., La Civita F., Ticconi C., Rago R., Campagnolo L. (2019). Molecular Signaling Regulating Endometrium-Blastocyst Crosstalk. Int. J. Mol. Sci..

[2] Blanco-Breindel M.F., Singh M., Kahn J. (2025). Endometrial Receptivity. StatPearls.

[3] Akaeda S., Aikawa S., Hirota Y. (2024). Spatial and molecular anatomy of the endometrium during embryo implantation: A current overview of key regulators of blastocyst invasion. FEBS J..

[4] Ma W.G., Song H., Das S.K., Paria B.C., Dey S.K. (2003). Estrogen is a critical determinant that specifies the duration of the window of uterine receptivity for implantation. Proc. Natl. Acad. Sci. USA.

[5] Ozdemir A.Z., Karli P., Gulumser C. (2022). Does high estrogen level negatively affect pregnancy success in frozen embryo transfer?. Arch. Med. Sci..

[6] Dorostghoal M., Ghaffari H.O., Marmazi F., Keikhah N. (2018). Overexpression of Endometrial Estrogen Receptor-Alpha in The Window of Implantation in Women with Unexplained Infertility. Int. J. Fertil. Steril..

[7] Gebril M., Hirota Y., Aikawa S., Fukui Y., Kaku T., Matsuo M., Hirata T., Akaeda S., Hiraoka T., Shimizu-Hirota R. (2020). Uterine Epithelial Progesterone Receptor Governs Uterine Receptivity Through Epithelial Cell Differentiation. Endocrinology.

[8] Hantak A.M., Bagchi I.C., Bagchi M.K. (2014). Role of uterine stromal-epithelial crosstalk in embryo implantation. Int. J. Dev. Biol..

[9] Ng S.W., Norwitz G.A., Pavlicev M., Tilburgs T., Simon C., Norwitz E.R. (2020). Endometrial Decidualization: The Primary Driver of Pregnancy Health. Int. J. Mol. Sci..

[10] Okada H., Tsuzuki T., Murata H. (2018). Decidualization of the human endometrium. Reprod. Med. Biol..

[11] Ramathal C.Y., Bagchi I.C., Taylor R.N., Bagchi M.K. (2010). Endometrial decidualization: Of mice and men. Semin. Reprod. Med..

[12] Ticconi C., Di Simone N., Campagnolo L., Fazleabas A. (2021). Clinical consequences of defective decidualization. Tissue Cell.

[13] Rawlings T.M., Makwana K., Tryfonos M., Lucas E.S. (2021). Organoids to model the endometrium: Implantation and beyond. Reprod. Fertil..

[14] Aplin J.D., Stevens A. (2022). Use of ’omics for endometrial timing: The cycle moves on. Hum. Reprod..

[15] Zhang S., Lin H., Kong S., Wang S., Wang H., Wang H., Armant D.R. (2013). Physiological and molecular determinants of embryo implantation. Mol. Aspects Med..

[16] Namiki T., Ito J., Kashiwazaki N. (2018). Molecular mechanisms of embryonic implantation in mammals: Lessons from the gene manipulation of mice. Reprod. Med. Biol..

[17] Chemerinski A., Liu C., Morelli S.S., Babwah A.V., Douglas N.C. (2022). Mouse Cre drivers: Tools for studying disorders of the human female neuroendocrine-reproductive axisdagger. Biol. Reprod..

[18] Benson G.V., Lim H., Paria B.C., Satokata I., Dey S.K., Maas R.L. (1996). Mechanisms of reduced fertility in *Hoxa-10* mutant mice: Uterine homeosis and loss of maternal Hoxa-10 expression. Development.

[19] Hsieh-Li H.M., Witte D.P., Weinstein M., Branford W., Li H., Small K., Potter S.S. (1995). *Hoxa 11* structure, extensive antisense transcription, and function in male and female fertility. Development.

[20] Stewart C.L., Kaspar P., Brunet L.J., Bhatt H., Gadi I., Kontgen F., Abbondanzo S.J. (1992). Blastocyst implantation depends on maternal expression of leukaemia inhibitory factor. Nature.

[21] Bilinski P., Roopenian D., Gossler A. (1998). Maternal IL-11Ralpha function is required for normal decidua and fetoplacental development in mice. Genes Dev..

[22] Robb L., Li R., Hartley L., Nandurkar H.H., Koentgen F., Begley C.G. (1998). Infertility in female mice lacking the receptor for interleukin 11 is due to a defective uterine response to implantation. Nat. Med..

[23] Lubahn D.B., Moyer J.S., Golding T.S., Couse J.F., Korach K.S., Smithies O. (1993). Alteration of reproductive function but not prenatal sexual development after insertional disruption of the mouse estrogen receptor gene. Proc. Natl. Acad. Sci. USA.

[24] Robker R.L., Hennebold J.D., Russell D.L. (2018). Coordination of Ovulation and Oocyte Maturation: A Good Egg at the Right Time. Endocrinology.

[25] Takeda K., Noguchi K., Shi W., Tanaka T., Matsumoto M., Yoshida N., Kishimoto T., Akira S. (1997). Targeted disruption of the mouse *Stat3* gene leads to early embryonic lethality. Proc. Natl. Acad. Sci. USA.

[26] Gurumurthy C.B., Lloyd K.C.K. (2019). Generating mouse models for biomedical research: Technological advances. Dis. Model. Mech..

[27] Mareckova M., Garcia-Alonso L., Moullet M., Lorenzi V., Petryszak R., Sancho-Serra C., Oszlanczi A., Icoresi Mazzeo C., Wong F.C.K., Kelava I. (2024). An integrated single-cell reference atlas of the human endometrium. Nat. Genet..

[28] Winkler I., Tolkachov A., Lammers F., Lacour P., Daugelaite K., Schneider N., Koch M.L., Panten J., Grunschlager F., Poth T. (2024). The cycling and aging mouse female reproductive tract at single-cell resolution. Cell.

[29] Branda C.S., Dymecki S.M. (2004). Talking about a revolution: The impact of site-specific recombinases on genetic analyses in mice. Dev. Cell.

[30] Soyal S.M., Mukherjee A., Lee K.Y., Li J., Li H., DeMayo F.J., Lydon J.P. (2005). Cre-mediated recombination in cell lineages that express the progesterone receptor. Genesis.

[31] Mukherjee A., Soyal S.M., Wheeler D.A., Fernandez-Valdivia R., Nguyen J., DeMayo F.J., Lydon J.P. (2006). Targeting iCre expression to murine progesterone receptor cell-lineages using bacterial artificial chromosome transgenesis. Genesis.

[32] Namiki T., Kamoshita M., Kageyama A., Terakawa J., Ito J., Kashiwazaki N. (2021). Utility of progesterone receptor-ires-Cre to generate conditional knockout mice for uterine study. Anim. Sci. J..

[33] Winuthayanon W., Hewitt S.C., Orvis G.D., Behringer R.R., Korach K.S. (2010). Uterine epithelial estrogen receptor alpha is dispensable for proliferation but essential for complete biological and biochemical responses. Proc. Natl. Acad. Sci. USA.

[34] Daikoku T., Ogawa Y., Terakawa J., Ogawa A., DeFalco T., Dey S.K. (2014). Lactoferrin-iCre: A new mouse line to study uterine epithelial gene function. Endocrinology.

[35] Uetzmann L., Burtscher I., Lickert H. (2008). A mouse line expressing *Foxa2*-driven Cre recombinase in node, notochord, floorplate, and endoderm. Genesis.

[36] Kelleher A.M., Allen C.C., Davis D.J., Spencer T.E. (2022). *Prss29* Cre recombinase mice are useful to study adult uterine gland function. Genesis.

[37] Bouchard M., Souabni A., Busslinger M. (2004). Tissue-specific expression of cre recombinase from the *Pax8* locus. Genesis.

[38] Contreras C.M., Akbay E.A., Gallardo T.D., Haynie J.M., Sharma S., Tagao O., Bardeesy N., Takahashi M., Settleman J., Wong K.K. (2010). *Lkb1* inactivation is sufficient to drive endometrial cancers that are aggressive yet highly responsive to mTOR inhibitor monotherapy. Dis. Model. Mech..

[39] Jamin S.P., Arango N.A., Mishina Y., Hanks M.C., Behringer R.R. (2002). Requirement of Bmpr1a for Mullerian duct regression during male sexual development. Nat. Genet..

[40] Cen C., Chen M., Zhou J., Zhang L., Duo S., Jiang L., Hou X., Gao F. (2020). Inactivation of *Wt1* causes pre-granulosa cell to steroidogenic cell transformation and defect of ovary developmentdagger. Biol. Reprod..

[41] Zheng W., Zhang H., Gorre N., Risal S., Shen Y., Liu K. (2014). Two classes of ovarian primordial follicles exhibit distinct developmental dynamics and physiological functions. Hum. Mol. Genet..

[42] Chung M.I., Bujnis M., Barkauskas C.E., Kobayashi Y., Hogan B.L.M. (2018). Niche-mediated BMP/SMAD signaling regulates lung alveolar stem cell proliferation and differentiation. Development.

[43] Dickson M.J., Oh Y., Gruzdev A., Li R., Balaguer N., Kelleher A.M., Spencer T.E., Wu S.P., DeMayo F.J. (2022). Inserting Cre recombinase into the Prolactin 8a2 gene for decidua-specific recombination in mice. Genesis.

[44] Holtwick R., Gotthardt M., Skryabin B., Steinmetz M., Potthast R., Zetsche B., Hammer R.E., Herz J., Kuhn M. (2002). Smooth muscle-selective deletion of guanylyl cyclase-A prevents the acute but not chronic effects of ANP on blood pressure. Proc. Natl. Acad. Sci. USA.

[45] Wendling O., Bornert J.M., Chambon P., Metzger D. (2009). Efficient temporally-controlled targeted mutagenesis in smooth muscle cells of the adult mouse. Genesis.

[46] Xin H.B., Deng K.Y., Rishniw M., Ji G., Kotlikoff M.I. (2002). Smooth muscle expression of Cre recombinase and eGFP in transgenic mice. Physiol. Genom..

[47] Cloud A.S., Koohestani F., McWilliams M.M., Ganeshkumar S., Gunewardena S., Graham A., Nothnick W.B., Chennathukuzhi V.M. (2022). Loss of the repressor REST affects progesterone receptor function and promotes uterine leiomyoma pathogenesis. Proc. Natl. Acad. Sci. USA.

[48] Ismail P.M., Li J., DeMayo F.J., O’Malley B.W., Lydon J.P. (2002). A novel LacZ reporter mouse reveals complex regulation of the progesterone receptor promoter during mammary gland development. Mol. Endocrinol..

[49] Medina-Laver Y., Rodriguez-Varela C., Salsano S., Labarta E., Dominguez F. (2021). What Do We Know about Classical and Non-Classical Progesterone Receptors in the Human Female Reproductive Tract? A Review. Int. J. Mol. Sci..

[50] Sun X., Bartos A., Whitsett J.A., Dey S.K. (2013). Uterine deletion of *Gp130* or *Stat3* shows implantation failure with increased estrogenic responses. Mol. Endocrinol..

[51] Lee K., Jeong J., Kwak I., Yu C.T., Lanske B., Soegiarto D.W., Toftgard R., Tsai M.J., Tsai S., Lydon J.P. (2006). Indian hedgehog is a major mediator of progesterone signaling in the mouse uterus. Nat. Genet..

[52] Daikoku T., Cha J., Sun X., Tranguch S., Xie H., Fujita T., Hirota Y., Lydon J., DeMayo F., Maxson R. (2011). Conditional deletion of Msx homeobox genes in the uterus inhibits blastocyst implantation by altering uterine receptivity. Dev. Cell.

[53] Franco H.L., Dai D., Lee K.Y., Rubel C.A., Roop D., Boerboom D., Jeong J.W., Lydon J.P., Bagchi I.C., Bagchi M.K. (2011). WNT4 is a key regulator of normal postnatal uterine development and progesterone signaling during embryo implantation and decidualization in the mouse. FASEB J..

[54] Jeong J.W., Lee H.S., Franco H.L., Broaddus R.R., Taketo M.M., Tsai S.Y., Lydon J.P., DeMayo F.J. (2009). beta-catenin mediates glandular formation and dysregulation of beta-catenin induces hyperplasia formation in the murine uterus. Oncogene.

[55] Yuan J., Cha J., Deng W., Bartos A., Sun X., Ho H.H., Borg J.P., Yamaguchi T.P., Yang Y., Dey S.K. (2016). Planar cell polarity signaling in the uterus directs appropriate positioning of the crypt for embryo implantation. Proc. Natl. Acad. Sci. USA.

[56] Kim T.H., Lee D.K., Franco H.L., Lydon J.P., Jeong J.W. (2010). ERBB receptor feedback inhibitor 1 regulation of estrogen receptor activity is critical for uterine implantation in mice. Biol. Reprod..

[57] Lee K.Y., Jeong J.W., Wang J., Ma L., Martin J.F., Tsai S.Y., Lydon J.P., DeMayo F.J. (2007). *Bmp2* is critical for the murine uterine decidual response. Mol. Cell. Biol..

[58] Hawkins S.M., Andreu-Vieyra C.V., Kim T.H., Jeong J.W., Hodgson M.C., Chen R., Creighton C.J., Lydon J.P., Gunaratne P.H., DeMayo F.J. (2012). Dysregulation of uterine signaling pathways in progesterone receptor-Cre knockout of dicer. Mol. Endocrinol..

[59] Kurihara I., Lee D.K., Petit F.G., Jeong J., Lee K., Lydon J.P., DeMayo F.J., Tsai M.J., Tsai S.Y. (2007). COUP-TFII mediates progesterone regulation of uterine implantation by controlling ER activity. PLoS Genet..

[60] Sun X., Terakawa J., Clevers H., Barker N., Daikoku T., Dey S.K. (2014). Ovarian LGR5 is critical for successful pregnancy. FASEB J..

[61] Marquardt R.M., Kim T.H., Yoo J.Y., Teasley H.E., Fazleabas A.T., Young S.L., Lessey B.A., Arora R., Jeong J.W. (2021). Endometrial epithelial ARID1A is critical for uterine gland function in early pregnancy establishment. FASEB J..

[62] Matsuo M., Yuan J., Kim Y.S., Dewar A., Fujita H., Dey S.K., Sun X. (2022). Targeted depletion of uterine glandular *Foxa2* induces embryonic diapause in mice. Elife.

[63] Parr B.A., McMahon A.P. (1998). Sexually dimorphic development of the mammalian reproductive tract requires *Wnt-7a*. Nature.

[64] Dunlap K.A., Filant J., Hayashi K., Rucker E.B., Song G., Deng J.M., Behringer R.R., DeMayo F.J., Lydon J., Jeong J.W. (2011). Postnatal deletion of Wnt7a inhibits uterine gland morphogenesis and compromises adult fertility in mice. Biol. Reprod..

[65] Stewart C.A., Wang Y., Bonilla-Claudio M., Martin J.F., Gonzalez G., Taketo M.M., Behringer R.R. (2013). CTNNB1 in mesenchyme regulates epithelial cell differentiation during Mullerian duct and postnatal uterine development. Mol. Endocrinol..

[66] Chi R.A., Xu X., Li J.L., Xu X., Hu G., Brown P., Willson C., Kirsanov O., Geyer C., Huang C.L. (2023). WNK1 is required during male pachynema to sustain fertility. iScience.

[67] Cheng J., Rosario G., Cohen T.V., Hu J., Stewart C.L. (2017). Tissue-Specific Ablation of the LIF Receptor in the Murine Uterine Epithelium Results in Implantation Failure. Endocrinology.

[68] Kelleher A.M., Peng W., Pru J.K., Pru C.A., DeMayo F.J., Spencer T.E. (2017). Forkhead box a2 (FOXA2) is essential for uterine function and fertility. Proc. Natl. Acad. Sci. USA.

[69] Yuan J., Deng W., Cha J., Sun X., Borg J.P., Dey S.K. (2018). Tridimensional visualization reveals direct communication between the embryo and glands critical for implantation. Nat. Commun..

[70] Cha J., Dey S.K. (2017). Hunting for Fox(A2): Dual roles in female fertility. Proc. Natl. Acad. Sci. USA.

[71] Kelleher A.M., Milano-Foster J., Behura S.K., Spencer T.E. (2018). Uterine glands coordinate on-time embryo implantation and impact endometrial decidualization for pregnancy success. Nat. Commun..

[72] Rizo J.A., Davenport K.M., Winuthayanon W., Spencer T.E., Kelleher A.M. (2023). Estrogen receptor alpha regulates uterine epithelial lineage specification and homeostasis. iScience.

[73] Fu D.J., De Micheli A.J., Bidarimath M., Ellenson L.H., Cosgrove B.D., Flesken-Nikitin A., Nikitin A.Y. (2020). Cells expressing PAX8 are the main source of homeostatic regeneration of adult mouse endometrial epithelium and give rise to serous endometrial carcinoma. Dis. Model. Mech..

[74] Suryo Rahmanto Y., Shen W., Shi X., Chen X., Yu Y., Yu Z.C., Miyamoto T., Lee M.H., Singh V., Asaka R. (2020). Inactivation of *Arid1a* in the endometrium is associated with endometrioid tumorigenesis through transcriptional reprogramming. Nat. Commun..

[75] Russo A., Czarnecki A.A., Dean M., Modi D.A., Lantvit D.D., Hardy L., Baligod S., Davis D.A., Wei J.J., Burdette J.E. (2018). PTEN loss in the fallopian tube induces hyperplasia and ovarian tumor formation. Oncogene.

[76] Granger K., Fitch S., Shen M., Lloyd J., Bhurke A., Hancock J., Ye X., Arora R. (2024). Murine uterine gland branching is necessary for gland function in implantation. Mol. Hum. Reprod..

[77] Mullen R.D., Ontiveros A.E., Moses M.M., Behringer R.R. (2019). AMH and AMHR2 mutations: A spectrum of reproductive phenotypes across vertebrate species. Dev. Biol..

[78] Huang C.C., Orvis G.D., Wang Y., Behringer R.R. (2012). Stromal-to-epithelial transition during postpartum endometrial regeneration. PLoS ONE.

[79] McCallum M.L., Pru C.A., Niikura Y., Yee S.P., Lydon J.P., Peluso J.J., Pru J.K. (2016). Conditional Ablation of Progesterone Receptor Membrane Component 1 Results in Subfertility in the Female and Development of Endometrial Cysts. Endocrinology.

[80] Robker R.L., Watson L.N., Robertson S.A., Dunning K.R., McLaughlin E.A., Russell D.L. (2014). Identification of sites of STAT3 action in the female reproductive tract through conditional gene deletion. PLoS ONE.

[81] Ghosh A., Syed S.M., Kumar M., Carpenter T.J., Teixeira J.M., Houairia N., Negi S., Tanwar P.S. (2020). In Vivo Cell Fate Tracing Provides No Evidence for Mesenchymal to Epithelial Transition in Adult Fallopian Tube and Uterus. Cell Rep..

[82] Dickson M.J., Gruzdev A., DeMayo F.J. (2023). iCre recombinase expressed in the anti-Mullerian hormone receptor 2 gene causes global genetic modification in the mousedagger. Biol. Reprod..

[83] Bellessort B., Bachelot A., Heude E., Alfama G., Fontaine A., Le Cardinal M., Treier M., Levi G. (2015). Role of *Foxl2* in uterine maturation and function. Hum. Mol. Genet..

[84] Zhang H., Risal S., Gorre N., Busayavalasa K., Li X., Shen Y., Bosbach B., Brannstrom M., Liu K. (2014). Somatic cells initiate primordial follicle activation and govern the development of dormant oocytes in mice. Curr. Biol..

[85] Xin Q., Yu G., Feng I., Dean J. (2023). Chromatin remodeling of prostaglandin signaling in smooth muscle enables mouse embryo passage through the female reproductive tract. Dev. Cell.

[86] Emons G., Mustea A., Tempfer C. (2020). Tamoxifen and Endometrial Cancer: A Janus-Headed Drug. Cancers.

[87] Wood C.E., Kaplan J.R., Fontenot M.B., Williams J.K., Cline J.M. (2010). Endometrial profile of tamoxifen and low-dose estradiol combination therapy. Clin. Cancer Res..

[88] Peri L.E., Koh B.H., Ward G.K., Bayguinov Y., Hwang S.J., Gould T.W., Mullan C.J., Sanders K.M., Ward S.M. (2015). A novel class of interstitial cells in the mouse and monkey female reproductive tracts. Biol. Reprod..

[89] Kirkwood P.M., Gibson D.A., Shaw I., Dobie R., Kelepouri O., Henderson N.C., Saunders P.T.K. (2022). Single-cell RNA sequencing and lineage tracing confirm mesenchyme to epithelial transformation (MET) contributes to repair of the endometrium at menstruation. Elife.

[90] Wattez J.S., Qiao L., Lee S., Natale D.R.C., Shao J. (2019). The platelet-derived growth factor receptor alpha promoter-directed expression of cre recombinase in mouse placenta. Dev. Dyn..

[91] Herring B.P., Hoggatt A.M., Burlak C., Offermanns S. (2014). Previously differentiated medial vascular smooth muscle cells contribute to neointima formation following vascular injury. Vasc. Cell.

[92] Wu S.P., Wang T., Yao Z.C., Peavey M.C., Li X., Zhou L., Larina I.V., DeMayo F.J. (2022). Myometrial progesterone receptor determines a transcription program for uterine remodeling and contractions during pregnancy. PNAS Nexus.

[93] Boretto M., Cox B., Noben M., Hendriks N., Fassbender A., Roose H., Amant F., Timmerman D., Tomassetti C., Vanhie A. (2017). Development of organoids from mouse and human endometrium showing endometrial epithelium physiology and long-term expandability. Development.

[94] Cindrova-Davies T., Zhao X., Elder K., Jones C.J.P., Moffett A., Burton G.J., Turco M.Y. (2021). Menstrual flow as a non-invasive source of endometrial organoids. Commun. Biol..

[95] Filby C.E., Wyatt K.A., Mortlock S., Cousins F.L., McKinnon B., Tyson K.E., Montgomery G.W., Gargett C.E. (2021). Comparison of Organoids from Menstrual Fluid and Hormone-Treated Endometrium: Novel Tools for Gynecological Research. J. Pers. Med..

[96] Turco M.Y., Gardner L., Hughes J., Cindrova-Davies T., Gomez M.J., Farrell L., Hollinshead M., Marsh S.G.E., Brosens J.J., Critchley H.O. (2017). Long-term, hormone-responsive organoid cultures of human endometrium in a chemically defined medium. Nat. Cell. Biol..

[97] Zhou W., Barton S., Cui J., Santos L.L., Yang G., Stern C., Kieu V., Teh W.T., Ang C., Lucky T. (2022). Infertile human endometrial organoid apical protein secretions are dysregulated and impair trophoblast progenitor cell adhesion. Front. Endocrinol..

[98] Zhang Y., Zhao R., Yang C., Song J., Liu P., Li Y., Liu B., Li T., Yin C., Lu M. (2024). Human Receptive Endometrial Organoid for Deciphering the Implantation Window.

[99] Kagawa H., Javali A., Khoei H.H., Sommer T.M., Sestini G., Novatchkova M., Scholte Op Reimer Y., Castel G., Bruneau A., Maenhoudt N. (2022). Human blastoids model blastocyst development and implantation. Nature.

[100] Corujo-Simon E., Bates L.E., Yanagida A., Jones K., Clark S., von Meyenn F., Reik W., Nichols J. (2024). Human trophectoderm becomes multi-layered by internalization at the polar region. Dev. Cell.

[101] Ahmad V., Yeddula S.G.R., Telugu B., Spencer T.E., Kelleher A.M. (2024). Development of Polarity-Reversed Endometrial Epithelial Organoids. Reproduction.

[102] Kleinova M., Varga I., Cehakova M., Valent M., Klein M. (2024). Exploring the black box of human reproduction: Endometrial organoids and assembloids-generation, implantation modeling, and future clinical perspectives. Front. Cell. Dev. Biol..

[103] Shibata S., Endo S., Nagai L.A.E., Kobayashi E.H., Oike A., Kobayashi N., Kitamura A., Hori T., Nashimoto Y., Nakato R. (2024). Modeling embryo-endometrial interface recapitulating human embryo implantation. Sci. Adv..

[104] Menche C., Farin H.F. (2021). Strategies for genetic manipulation of adult stem cell-derived organoids. Exp. Mol. Med..

[105] Guo C., Ma X., Gao F., Guo Y. (2023). Off-target effects in CRISPR/Cas9 gene editing. Front. Bioeng. Biotechnol..

[106] Syed S.M., Kumar M., Ghosh A., Tomasetig F., Ali A., Whan R.M., Alterman D., Tanwar P.S. (2020). Endometrial Axin2(+) Cells Drive Epithelial Homeostasis, Regeneration, and Cancer following Oncogenic Transformation. Cell Stem Cell.

[107] Vitorino R. (2024). Transforming Clinical Research: The Power of High-Throughput Omics Integration. Proteomes.

[108] Huang J., Qin H., Yang Y., Chen X., Zhang J., Laird S., Wang C.C., Chan T.F., Li T.C. (2017). A comparison of transcriptomic profiles in endometrium during window of implantation between women with unexplained recurrent implantation failure and recurrent miscarriage. Reproduction.

[109] Aljubran F., Nothnick W.B. (2021). Long non-coding RNAs in endometrial physiology and pathophysiology. Mol. Cell. Endocrinol..

[110] Wang C., Lin Y., Li S., Guan J. (2024). Deconvolution from bulk gene expression by leveraging sample-wise and gene-wise similarities and single-cell RNA-Seq data. BMC Genom..

[111] Garcia-Alonso L., Handfield L.F., Roberts K., Nikolakopoulou K., Fernando R.C., Gardner L., Woodhams B., Arutyunyan A., Polanski K., Hoo R. (2021). Mapping the temporal and spatial dynamics of the human endometrium in vivo and in vitro. Nat. Genet..

[112] Walker E.R., McGrane M., Aplin J.D., Brison D.R., Ruane P.T. (2023). A systematic review of transcriptomic studies of the human endometrium reveals inconsistently reported differentially expressed genes. Reprod. Fertil..

[113] Liu S., Li X., Gu Z., Wu J., Jia S., Shi J., Dai Y., Wu Y., Yan H., Zhang J. (2025). Single-cell and spatial transcriptomic profiling revealed niche interactions sustaining growth of endometriotic lesions. Cell Genom..

[114] Lai Z.Z., Wang Y., Zhou W.J., Liang Z., Shi J.W., Yang H.L., Xie F., Chen W.D., Zhu R., Zhang C. (2022). Single-cell transcriptome profiling of the human endometrium of patients with recurrent implantation failure. Theranostics.

[115] Krjutskov K., Katayama S., Saare M., Vera-Rodriguez M., Lubenets D., Samuel K., Laisk-Podar T., Teder H., Einarsdottir E., Salumets A. (2016). Single-cell transcriptome analysis of endometrial tissue. Hum. Reprod..

[116] Bunis D.G., Wang W., Vallve-Juanico J., Houshdaran S., Sen S., Ben Soltane I., Kosti I., Vo K.C., Irwin J.C., Giudice L.C. (2021). Whole-Tissue Deconvolution and scRNAseq Analysis Identify Altered Endometrial Cellular Compositions and Functionality Associated With Endometriosis. Front. Immunol..

[117] Koot Y.E., Teklenburg G., Salker M.S., Brosens J.J., Macklon N.S. (2012). Molecular aspects of implantation failure. Biochim. Biophys. Acta.

[118] Yang Y., Zhu Q.Y., Liu J.L. (2021). Deciphering mouse uterine receptivity for embryo implantation at single-cell resolution. Cell Prolif..

[119] Yang Y., He J.P., Liu J.L. (2021). Cell-Cell Communication at the Embryo Implantation Site of Mouse Uterus Revealed by Single-Cell Analysis. Int. J. Mol. Sci..

[120] Jiang L., Cao D., Yeung W.S.B., Lee K.F. (2022). Single-Cell RNA-Sequencing Reveals Interactions between Endometrial Stromal Cells, Epithelial Cells, and Lymphocytes during Mouse Embryo Implantation. Int. J. Mol. Sci..

[121] Cable D.M., Murray E., Zou L.S., Goeva A., Macosko E.Z., Chen F., Irizarry R.A. (2022). Robust decomposition of cell type mixtures in spatial transcriptomics. Nat. Biotechnol..

[122] Cao D., Liu Y., Cheng Y., Wang J., Zhang B., Zhai Y., Zhu K., Liu Y., Shang Y., Xiao X. (2025). Time-series single-cell transcriptomic profiling of luteal-phase endometrium uncovers dynamic characteristics and its dysregulation in recurrent implantation failures. Nat. Commun..

[123] Li R., Wang T.Y., Xu X., Emery O.M., Yi M., Wu S.P., DeMayo F.J. (2022). Spatial transcriptomic profiles of mouse uterine microenvironments at pregnancy day 7.5dagger. Biol. Reprod..

[124] Yang M., Ong J., Meng F., Zhang F., Shen H., Kitt K., Liu T., Tao W., Du P. (2023). Spatiotemporal insight into early pregnancy governed by immune-featured stromal cells. Cell.

[125] Retis-Resendiz A.M., Gonzalez-Garcia I.N., Leon-Juarez M., Camacho-Arroyo I., Cerbon M., Vazquez-Martinez E.R. (2021). The role of epigenetic mechanisms in the regulation of gene expression in the cyclical endometrium. Clin. Epigenetics.

[126] Gujral P., Mahajan V., Lissaman A.C., Ponnampalam A.P. (2020). Histone acetylation and the role of histone deacetylases in normal cyclic endometrium. Reprod. Biol. Endocrinol..

[127] Munro S.K., Farquhar C.M., Mitchell M.D., Ponnampalam A.P. (2010). Epigenetic regulation of endometrium during the menstrual cycle. Mol. Hum. Reprod..

[128] Monteiro J.B., Colon-Diaz M., Garcia M., Gutierrez S., Colon M., Seto E., Laboy J., Flores I. (2014). Endometriosis is characterized by a distinct pattern of histone 3 and histone 4 lysine modifications. Reprod. Sci..

[129] Samadieh Y., Favaedi R., Ramezanali F., Afsharian P., Aflatoonian R., Shahhoseini M. (2019). Epigenetic Dynamics of *HOXA10* Gene in Infertile Women With Endometriosis. Reprod. Sci..

[130] Taylor H.S., Arici A., Olive D., Igarashi P. (1998). HOXA10 is expressed in response to sex steroids at the time of implantation in the human endometrium. J. Clin. Investig..

[131] Skene P.J., Henikoff S. (2017). An efficient targeted nuclease strategy for high-resolution mapping of DNA binding sites. Elife.

[132] Mumbach M.R., Rubin A.J., Flynn R.A., Dai C., Khavari P.A., Greenleaf W.J., Chang H.Y. (2016). HiChIP: Efficient and sensitive analysis of protein-directed genome architecture. Nat. Methods.

[133] Mumbach M.R., Satpathy A.T., Boyle E.A., Dai C., Gowen B.G., Cho S.W., Nguyen M.L., Rubin A.J., Granja J.M., Kazane K.R. (2017). Enhancer connectome in primary human cells identifies target genes of disease-associated DNA elements. Nat. Genet..

[134] Barral A., Dejardin J. (2023). The chromatin signatures of enhancers and their dynamic regulation. Nucleus.

[135] Vrljicak P., Lucas E.S., Lansdowne L., Lucciola R., Muter J., Dyer N.P., Brosens J.J., Ott S. (2018). Analysis of chromatin accessibility in decidualizing human endometrial stromal cells. FASEB J..

[136] Vrljicak P., Lucas E.S., Tryfonos M., Muter J., Ott S., Brosens J.J. (2023). Dynamic chromatin remodeling in cycling human endometrium at single-cell level. Cell. Rep..

[137] Martinez-Sarmiento J.A., Cosma M.P., Lakadamyali M. (2024). Dissecting gene activation and chromatin remodeling dynamics in single human cells undergoing reprogramming. Cell. Rep..

[138] Ye L., Dimitriadis E. (2025). Endometrial Receptivity-Lessons from “Omics”. Biomolecules.

[139] Yang J., Wang L., Ma J., Diao L., Chen J., Cheng Y., Yang J., Li L. (2023). Endometrial proteomic profile of patients with repeated implantation failure. Front. Endocrinol..

[140] Guo X., Li T.C., Chen X. (2021). The endometrial proteomic profile around the time of embryo implantationdagger. Biol. Reprod..

[141] Molina N.M., Jurado-Fasoli L., Sola-Leyva A., Sevilla-Lorente R., Canha-Gouveia A., Ruiz-Duran S., Fontes J., Aguilera C.M., Altmae S. (2023). Endometrial whole metabolome profile at the receptive phase: Influence of Mediterranean Diet and infertility. Front. Endocrinol..

[142] Mancini V., Schrimpe-Rutledge A.C., Codreanu S.G., Sherrod S.D., McLean J.A., Picton H.M., Pensabene V. (2021). Metabolomic Analysis Evidences That Uterine Epithelial Cells Enhance Blastocyst Development in a Microfluidic Device. Cells.

[143] Reschini M., Benaglia L., Ceriotti F., Borroni R., Ferrari S., Castiglioni M., Guarneri D., Porcaro L., Vigano P., Somigliana E. (2022). Endometrial microbiome: Sampling, assessment, and possible impact on embryo implantation. Sci. Rep..

[144] Lozano F.M., Lledo B., Morales R., Cascales A., Hortal M., Bernabeu A., Bernabeu R. (2023). Characterization of the Endometrial Microbiome in Patients with Recurrent Implantation Failure. Microorganisms.

